# Molecular epidemiology and emerging *tet*(X)-associated resistance of *Elizabethkingia* spp. in Taiwan, 2016–2022

**DOI:** 10.1128/aac.00387-26

**Published:** 2026-06-12

**Authors:** Yu-Tsung Huang, Cheng-Yen Kao, Han-Yueh Kuo, Li-Ting Kao, Yu-Chi Chang, Yen-Ting Liao, Li-Cheng Wu, Tzu-Ling Chen, Yu-Kai Tu, Tsung-Ying Yang

**Affiliations:** 1 Department of Laboratory Medicine, National Taiwan University Hospital, National Taiwan University College of Medicine 38005, Taipei, Taiwan; 2 Institute of Microbiology and Immunology, College of Life Science, National Yang Ming Chiao Tung University 535308, Taipei, Taiwan; 3 Division of Infectious Disease, National Taiwan University Hospital Hsin-Chu Branch, Hsin-chu, Taiwan; 4 Department of Internal Medicine, National Taiwan University Hospital Hsin-Chu Branch, Hsin-chu, Taiwan; 5 Graduate Institute of Medicine, College of Medicine, Kaohsiung Medical University 164790, Kaohsiung, Taiwan; 6 Department of Medical Laboratory and Regenerative Medicine, MacKay Medical University 145474, New Taipei City, Taiwan; 7 Institute of Biomedical Science, MacKay Medical University 145474, New Taipei City, Taiwan; 8 Department of Medical Laboratory Science, I-Shou University 54791, Kaohsiung, Taiwan; 9 Research Institute for Science and Engineering, Waseda University 13148, Tokyo, Japan; 10 Department of Medical Laboratory Science and Biotechnology, Kaohsiung Medical University 38023, Kaohsiung, Taiwan; University of Fribourg, Fribourg, Switzerland

**Keywords:** *Elizabethkingia*, antimicrobial resistance, *tet*(X), fluoroquinolone resistance, tetracycline resistance

## Abstract

*Elizabethkingia* species are opportunistic gram-negative pathogens associated with nosocomial infections and limited therapeutic options. Longitudinal data characterizing antimicrobial resistance and molecular determinants in Taiwan remain scarce. A total of 191 non-duplicate *Elizabethkingia* clinical isolates were collected at the National Taiwan University Hospital between 2016 and 2022. Species identification was confirmed by 16S rRNA sequencing. *Elizabethkingia anophelis* predominated (81.7%), followed by *E. meningoseptica* (9.9%) and *E. miricola* (7.9%). No major clone was identified. Genes involved in sialic acid metabolism were detected as follows: *glmS* in 93.2% (178/191), *glmM* in 97.4% (186/191), *nagB* in 98.4% (188/191), *neuC2* in 24.5% (46/191), and *neuC* in 2.1% (4/191) of isolates. Antimicrobial susceptibility testing showed very high nonsusceptibility for most agents (≥99.5%). Lower rates of nonsusceptibility were observed for levofloxacin (MIC_50_/MIC_90_ = 1/>8 µg/mL; 68.6% susceptible), ciprofloxacin (2/>4 µg/mL; 49.2%), tigecycline (4/>8 µg/mL; 23.0%), and rifampin (>8/>16 µg/mL; 19.9%). Using the breakpoint for other non-Enterobacterales, minocycline was active against all isolates (100.0%, 191/191); however, applying the *S. maltophilia* criteria, 42 isolates (22.0%) were nonsusceptible to minocycline. Among tetracycline determinants, *tet*(X) was detected in 22 isolates (11.5%) and *tet*(K) in 2 isolates (1.0%). With the criteria of *S. maltophilia*, 10 *tet*(X)-positive isolates (10/22, 45.5%) showed non-susceptibility to minocycline, implying that *tet*(X) may contribute to the elevated MIC of minocycline. Our surveillance revealed a rapid increase in resistance to fluoroquinolones and rifampin and the emergence of *tet*(X)-mediated tetracycline resistance in *Elizabethkingia* spp. These findings underscore the need for continued molecular monitoring and region-specific antimicrobial stewardship.

## INTRODUCTION

*Elizabethkingia* species are emerging opportunistic pathogens increasingly recognized for causing severe healthcare-associated infections, particularly in immunocompromised patients. They are intrinsically multidrug-resistant and associated with high mortality rates in nosocomial pneumonia, meningitis, bloodstream, and wound infections (1, 2). In Taiwan, several studies have highlighted the clinical significance, showing their infections are linked to intensive care units in vulnerable populations, and the potential for nosocomial transmission (1, 2). However, the treatment of *Elizabethkingia* infections remains highly challenging due to the intrinsic resistance to most β-lactams, which is mediated by chromosomally encoded metallo-β-lactamases, such as *bla*_B_, *bla*_GOB_, and *bla*_CME_, which are widely distributed among clinical isolates (3, 4). According to previous surveillance in Taiwan, the universal resistance of *Elizabethkingia* spp. to cephalosporins and carbapenems has been observed, underscoring the clinical limitations of these antibiotics (5, 6).

Several epidemiological studies have defined the clinical burden and molecular traits of *Elizabethkingia* infections. A multicenter analysis reported rising rates of fluoroquinolone-nonsusceptibility in *Elizabethkingia meningoseptica* bacteremia, especially in Northern Taiwan (7). Another study found high mortality (24%–60%) associated with *Elizabethkingia anophelis* bacteremia in a tertiary center, with antibiotic resistance complicating treatment (8–13). Molecular characterization of isolates revealed diverse quinolone-resistance-determining region (QRDR) mutations in *gyrA* at codon Ser83 (e.g., Ser83Ile or Ser83Arg) and *gyrB* at codon Ser464, as well as in *parC* at codon Ser80 (14). In environmental and hospital contexts, genomic sequencing of isolates (e.g., EM361-97 from Taiwan) illustrated high genetic diversity and multiple resistance determinants (15). Taiwan’s antimicrobial surveillance also highlighted minocycline’s relatively preserved effectiveness (>90% susceptibility), contrasting with poor rifampin and fluoroquinolone activity (6). By contrast, tetracycline resistance in *Elizabethkingia* is less uniformly distributed but increasingly recognized as a threat due to the dissemination of *tet* genes, especially the *tet*(X) family (16). The flavin-dependent monooxygenase *tet*(X) can enzymatically inactivate tigecycline and related tetracyclines, with variants such as *tet*(X3) and *tet*(X4) reported in environmental and clinical isolates (16–18). Other mechanisms, including ribosomal protection proteins and efflux pumps, have been described in related gram-negative bacteria but appear less common in *Elizabethkingia* (19). To date, however, the prevalence of *tet*(X) and related determinants in Taiwan remains undefined, highlighting the importance of surveillance studies to clarify the contribution of these resistance mechanisms in local clinical settings.

Despite these investigations, most studies have short-term or outbreak-focused designs. There remains a need for prolonged surveillance to understand evolving antimicrobial resistance patterns over time. This study sought to analyze 191 clinical *Elizabethkingia* blood isolates from a Taiwanese medical center over 7 years (2016–2022), focusing on temporal trends in antimicrobial susceptibility, QRDR mutations related to fluoroquinolones, and resistance determinants for tetracycline and β-lactams.

## RESULTS

### Characterization of *Elizabethkingia* spp. in this study

*E. anophelis* was most common (*n* = 156, 81.7%), followed by *E. meningoseptica* (*n* = 19, 9.9%) and *E. miricola* (*n* = 15, 7.9%); a single *Elizabethkingia ursingii* isolate was also recovered (0.5%). The predominance of *E. anophelis* was consistent year to year, accounting for 73.1%–93.8% of annual isolates (Table 1; Fig. S1). PCR screening showed that the three intrinsic β-lactamases (*bla*_GOB_, *bla*_B_, and *bla*_CME_) were present in nearly all *E. anophelis* isolates. The detection rates were 94.9% (148/156) for *bla*_GOB_, 97.4% (152/156) for *bla*_B_, and 99.4% (155/156) for *bla*_CME_ (Table S3). Overall, *bla*_CME_ was the most consistently detected β-lactamase across the 191 isolates (97.9%). The single *E. ursingii* isolate also carried all three genes.

**TABLE 1:** Years of clinical isolation of *Elizabethkingia* spp

Species, *n/N (%*)	Year							Total
	2016	2017	2018	2019	2020	2021	2022	
*E. anophelis*	30 (93.8)	28 (84.8)	22 (78.6)	16 (80)	23 (82.1)	18 (75)	19 (73.1)	156 (81.7)
*E. meningoseptica*	2 (6.3)	3 (9.1)	4 (14.3)	1 (5)	3 (10.7)	3 (12.5)	3 (11.5)	19 (9.9)
*E. miricola*	0 (0)	1 (3)	2 (7.1)	3 (15)	2 (7.1)	3 (12.5)	4 (15.4)	15 (7.9)
*E. ursingii*	0 (0)	1 (3)	0 (0)	0 (0)	0 (0)	0 (0)	0 (0)	1 (0.5)
Total	**32**	**33**	**28**	**20**	**28**	**24**	**26**	**191**

We examined several virulence-associated genes linked to sialic-acid synthesis, transport, and utilization (Table S4). Genes involved in amino-sugar metabolism were widely present: *glmS*, *glmM*, and *nagB* appeared in over 90% of isolates, while *neuC2* was detected in about one-quarter and *neuC* in only four isolates, all belonging to *E. meningoseptica*. These distributions suggest that pathways supporting amino-sugar and sialic-acid utilization are broadly conserved, whereas *de novo* sialic-acid synthesis is limited to specific lineages. The pattern is consistent with gene function: *glmS*, *glmM*, and *nagB* participate in central metabolic routes essential for cell-wall and glycan biosynthesis, while *neuC* and *neuC2* encode epimerases associated with a more specialized sialylation capacity. Overall, NTUH isolates collected over 7 years show stable conservation of core amino-sugar metabolism with species-dependent acquisition of sialic-acid synthesis genes (Table S4) (20).

The banding patterns of PFGE using *Xho*I restriction digestion demonstrated high genetic diversity among isolates (Fig. S2 to S4). In *E. anophelis*, we identified two large clusters, each containing seven isolates (7/156). In contrast, the remaining isolates and the other two *Elizabethkingia* species showed high genetic diversity. This observation indicates that infections were largely caused by genetically distinct strains rather than the expansion of a single epidemic clone, agreeing with prior genomic observations (14). Clinically, the widespread presence of these loci may help explain the organism’s ability to persist on mucosal surfaces and medical devices, suggesting the need for contact/water system control measures when clusters arise (21, 22).

### Antimicrobial susceptibility testing

**Fig 1:**
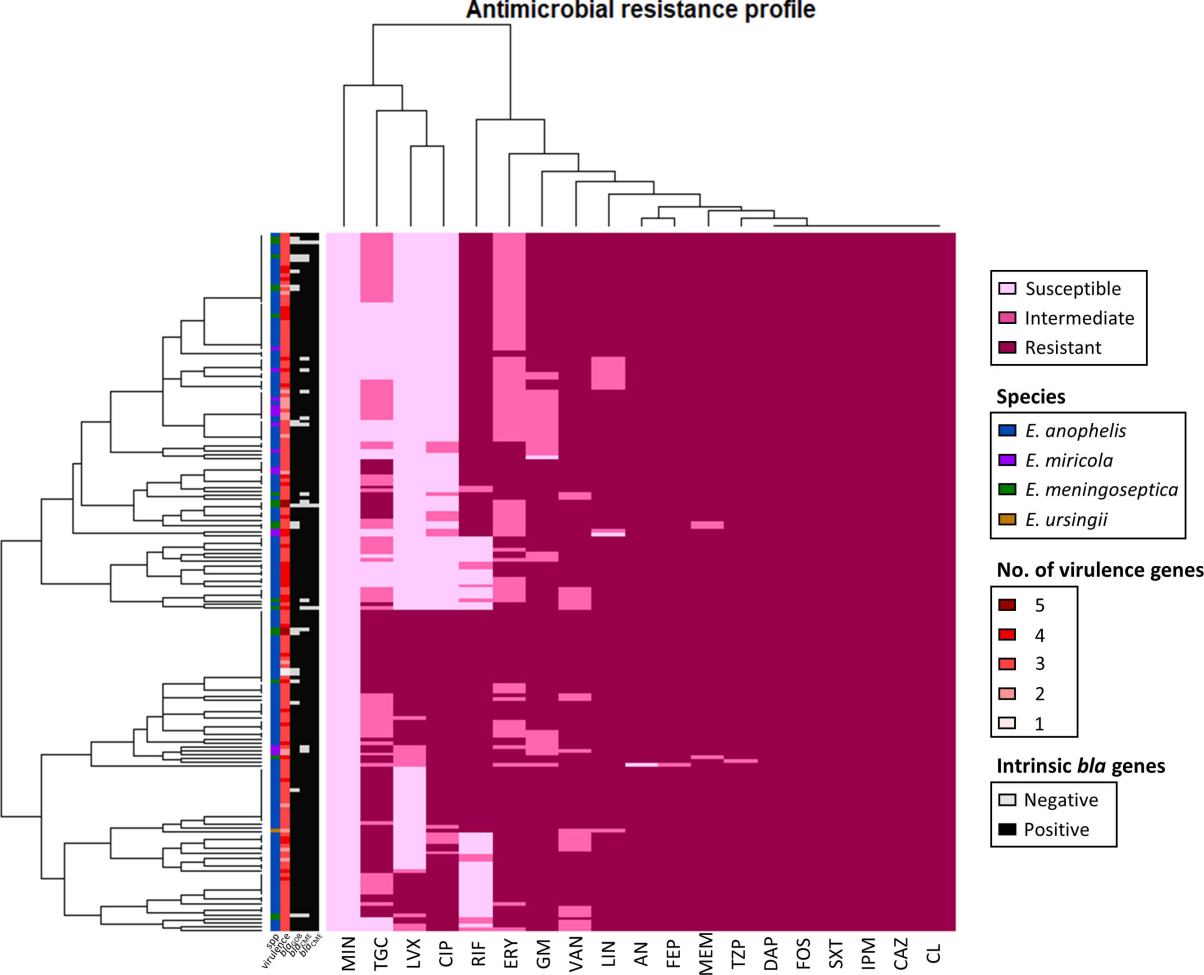
Heatmap of antimicrobial-resistant profiles in 191 *Elizabethkingia* isolates. MIN, minocycline; TGC, tigecycline; LEV, levofloxacin; CIP, ciprofloxacin; RIF, rifmapin; ERY, erythromycin; GM, gentamicin; VAN, vancomycin; LIN, linezolid; AN, amikacin; FEP, cefepime; MEM, meropenem; TZP, piperacillin-tazobactam; DAP, daptomycin; FOS, fosfomycin; SXT, trimethoprim/sulfamethoxazole; IMP, Imipenem; CAZ, ceftazidime; CL, colistin. Antimicrobial agents are shown across the horizontal axis, with color coding to denote susceptibility categories: light pink for susceptible, pink for intermediate resistance, and dark red for resistant. The dendrogram on the left clusters the isolates based on the similarity of their antimicrobial resistance patterns.

Among the 191 *Elizabethkingia* isolates, resistance profiles across 19 antibiotics varied widely (Table 2). MIC distributions for the five agents with susceptibilities, rifampin, minocycline, ciprofloxacin, levofloxacin, and tigecycline, are shown in Table 3. Minocycline demonstrated the most favorable activity, with an MIC range of 0.25–4 μg/mL, MIC_50_ of 1 μg/mL, and MIC_90_ of 2 μg/mL. Both fluoroquinolones showed broader distributions: levofloxacin (0.5–>8 µg/mL; MIC_50_ = 1 µg/mL; MIC_90_ >8 µg/mL) and ciprofloxacin (0.5–>4 µg/mL; MIC_50_ = 2 µg/mL; MIC_90_ >4 µg/mL). Rifampin and tigecycline displayed consistently elevated MIC values; rifampin had an MIC range of 1–>16 µg/mL with MIC_50_ >8 µg/mL and MIC_90_ >16 µg/mL, while tigecycline ranged from 0.5–>8 µg/mL with MIC_50_ = 4 µg/mL and MIC_90_ >8 µg/mL. A cluster of 19 isolates (17 *E. anophelis*, 2 *E. meningoseptica*) showed pandrug resistance to 18 agents (Fig. 1), yet PFGE clustering suggested heterogeneity without clear clonal expansion (Fig. S2 to S4).

**TABLE 2:** Antimicrobial susceptibility testing of *Elizabethkingia* spp^*b*^

Antibiotics^*a*^	Interpretation of susceptibility, *n/N (%*)											
	*E. anophelis* (156)			*E. meningseptica* (19)			*E. miricola* (15)			Total (191)^*a*^		
	S	I	R	S	I	R	S	I	R	S	I	R
Ceftazidime	0 (0)	0 (0)	156 (100)	0 (0)	0 (0)	19 (100)	0 (0)	0 (0)	15 (100)	0 (0)	0 (0)	191 (100)
Cefepime	0 (0)	1 (0.6)	155 (99.4)	0 (0)	0 (0)	19 (100)	0 (0)	0 (0)	15 (100)	0 (0)	1 (0.5)	190 (99.5)
Imipenem	0 (0)	0 (0)	156 (100)	0 (0)	0 (0)	19 (100)	0 (0)	0 (0)	15 (100)	0 (0)	0 (0)	191 (100)
Meropenem	0 (0)	0 (0)	156 (100)	0 (0)	3 (15.8)	16 (84.2)	0 (0)	0 (0)	15 (100)	0 (0)	3 (1.6)	188 (98.4)
Piperacillin-Tazobactam	0 (0)	1 (0.6)	155 (99.4)	0 (0)	0 (0)	19 (100)	0 (0)	0 (0)	15 (100)	0 (0)	1 (0.5)	190 (99.5)
Gentamicin	1 (0.6)	22 (14.1)	133 (85.3)	0 (0)	0 (0)	19 (100)	0 (0)	9 (60)	6 (40)	1 (0.5)	31 (16.2)	159 (83.2)
Amikacin	1 (0.6)	0 (0)	155 (99.4)	0 (0)	0 (0)	19 (100)	0 (0)	0 (0)	15 (100)	1 (0.5)	0 (0)	190 (99.5)
Ciprofloxacin	73 (46.8)	10 (6.4)	73 (46.8)	12 (63.2)	1 (5.3)	6 (31.6)	9 (60)	3 (20)	3 (20)	94 (49.2)	14 (7.3)	83 (43.5)
Levofloxacin	105 (67.3)	6 (3.8)	45 (28.8)	13 (68.4)	2 (10.5)	4 (21.1)	12 (80)	3 (20)	0 (0)	131 (68.6)	11 (5.8)	49 (25.7)
Minocycline	156 (100)	0 (0)	0 (0)	19 (100)	0 (0)	0 (0)	15 (100)	0 (0)	0 (0)	191 (100)	0 (0)	0 (0)
Tigecycline	36 (23.1)	59 (37.8)	61 (39.1)	2 (10.5)	9 (47.4)	8 (42.1)	6 (40)	5 (33.3)	4 (26.7)	44 (23)	73 (38.2)	74 (38.7)
Vancomycin	0 (0)	17 (10.9)	139 (89.1)	0 (0)	4 (21.1)	15 (78.9)	0 (0)	1 (6.7)	14 (93.3)	0 (0)	23 (12)	168 (88)
Rifampin	36 (23.1)	9 (5.8)	111 (71.2)	2 (10.5)	2 (10.5)	15 (78.9)	0 (0)	0 (0)	15 (100)	38 (19.9)	11 (5.8)	142 (74.3)
Sulfamethoxazole-Trimethoprim	0 (0)	0 (0)	156 (100)	0 (0)	0 (0)	19 (100)	0 (0)	0 (0)	15 (100)	0 (0)	0 (0)	191 (100)
Linezolid	0 (0)	8 (5.1)	148 (94.9)	0 (0)	0 (0)	19 (100)	1 (6.7)	2 (13.3)	12 (80)	1 (0.5)	11 (5.8)	179 (93.7)
Fosfomycin	0 (0)	0 (0)	156 (100)	0 (0)	0 (0)	19 (100)	0 (0)	0 (0)	15 (100)	0 (0)	0 (0)	191 (100)
Daptomycin	0 (0)	0 (0)	156 (100)	0 (0)	0 (0)	19 (100)	0 (0)	0 (0)	15 (100)	0 (0)	0 (0)	191 (100)
Erythromycin	0 (0)	66 (42.3)	90 (57.7)	0 (0)	11 (57.9)	8 (42.1)	0 (0)	10 (66.7)	5 (33.3)	0 (0)	87 (45.5)	104 (54.5)
Colistin	0 (0)	0 (0)	156 (100)	0 (0)	0 (0)	19 (100)	0 (0)	0 (0)	15 (100)	0 (0)	0 (0)	191 (100)

^*a*^ One of the isolates we tested is *E. ursingii*.

^*b*^ S, susceptible; I, intermediate; R, resistant.

**TABLE 3:** MIC distribution of selected antimicrobials against *Elizabethkingia* spp

Species		Rifampin	Minocycline	Ciprpfloxacin	Levofloxacin	Tigecycline
*E. anophelis* (156)	Susceptibility	36 (23.1)	156 (100)	73 (46.8)	105 (67.3)	36 (23.1)
	MIC_50_	8	1	2	1	4
	MIC_90_	16	2	>4	>8	>8
	MIC range	0.125 - > 16	0.125–4	0.125 - > 4	0.25 - > 8	0.5 - > 8
*E. meningoseptica* (19)	susceptibility	2 (10.5)	19 (100)	12 (63.2)	13 (68.4)	2 (10.5)
	MIC_50_	8	0.5	0.5	0.5	4
	MIC_90_	16	1	>4	>8	>8
	MIC range	0.25–16	0.25–1	0.125 - > 4	0.25 - > 8	0.5 - > 8
*E. miricola* (15)	Susceptibility	0 (0)	15 (100)	9 (60)	12 (80)	6 (40)
	MIC_50_	16	0.5	1	1	4
	MIC_90_	>16	1	>4	4	>8
	MIC range	2 - > 16	0.125–2	0.5 - > 4	0.25–4	0.5 - > 8
Total (191)	Susceptibility	38 (19.9)	191 (100)	94 (49.2)	131 (68.6)	44 (23)
	MIC_50_	8	1	2	1	4
	MIC_90_	16	2	>4	>8	>8
	MIC range	0.125 - > 16	0.25–4	0.125 - > 4	0.25 - > 8	0.5 - > 8

**Fig 2:**
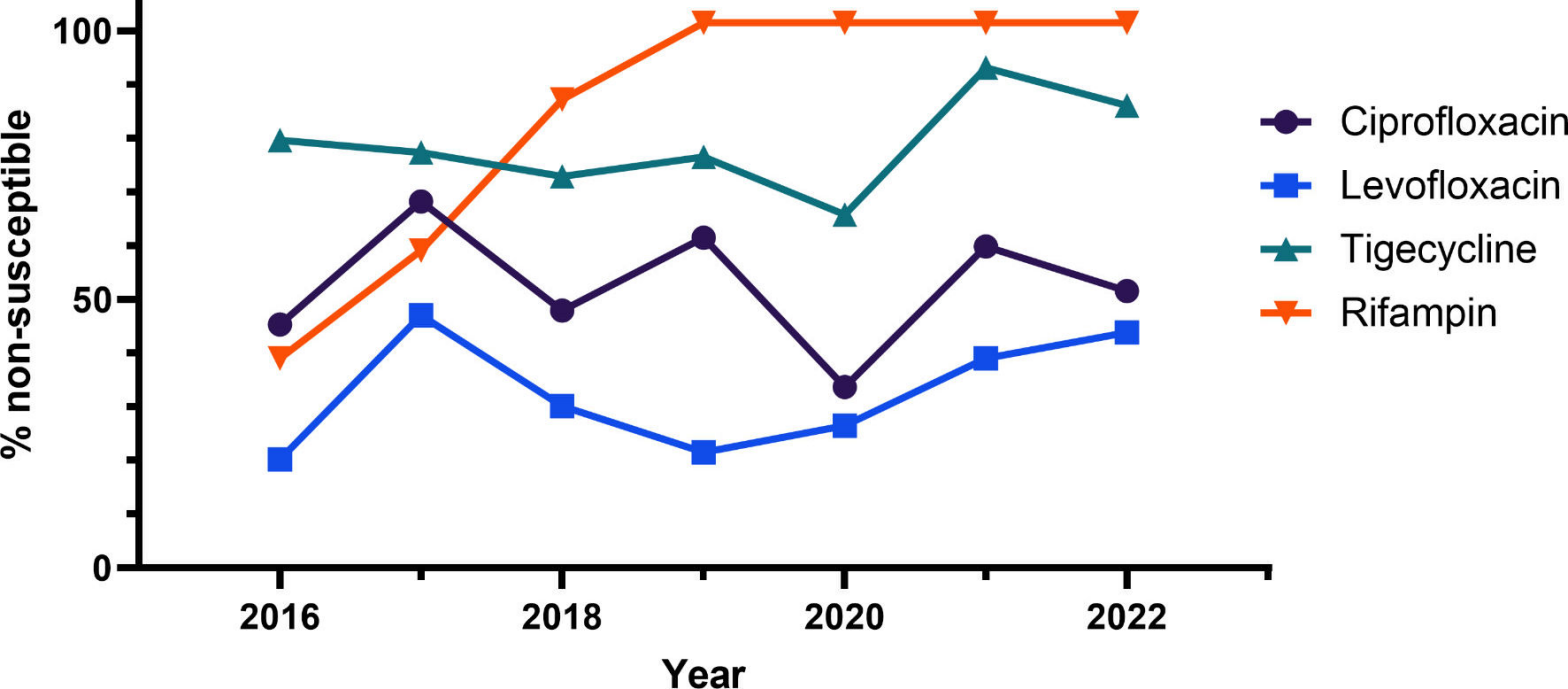
Trends of resistant rates for ciprofloxacin, levofloxacin, tigecycline, and rifampin over 7 years.

Resistance trends from 2016 to 2022 (Fig. 2) showed fluctuating ciprofloxacin nonsusceptibility, ranging from 43.8% (14/32) to 66.7% (22/33), without a significant temporal trend (slope −0.4429, *P* = 0.9997). Levofloxacin nonsusceptibility rose from 18.7% (6/32) to 42.3% (11/26) though this overall trend did not reach statistical significance (slope +1.8286, *P* = 0.0552). However, after excluding the spike in 2017, a significant upward trend was observed (slope +3.6357, *P* = 0.0341; data not shown). Tigecycline nonsusceptibility remained high (64.3%–91.6%) and demonstrated a significant upward slope (+1.5714, *P* = 0.0036). Rifampin resistance increased sharply from 37.5% to 100% after 2019, with a significant trend (slope +10.236, *P* = 0.0005), indicating rapid loss of efficacy.

### Fluoroquinolone-resistant mechanisms

Since levofloxacin is a third-generation fluoroquinolone and its resistance patterns in our study completely overlapped with those of ciprofloxacin, we focused our mutation analysis on the 96 ciprofloxacin-non-susceptible isolates. This approach allowed us to more effectively identify the specific genetic changes responsible for the transition from ciprofloxacin resistance to broader levofloxacin resistance. Among the 96 ciprofloxacin-nonsusceptible isolates (83 *E. anophelis*, 7 *E. meningoseptica*, 6 *E. miricola*), amino-acid substitutions were identified across *gyrA*, *gyrB*, *parC*, and *parE* (Table 4). In *gyrA*, position 83 remained the principal hotspot: Ser83Ile was detected in 36 isolates (37.5%), Ser83Arg in 6 (6.3%), and a total of 44 isolates carried substitutions at this position. Two isolates also harbored Phe217Pro. In *gyrB*, Gly284Ser was the most common substitution (10 isolates, 10.4%), with several less frequent variants, including Gly283Glu (3 isolates) and combinations involving positions 283, 284, and 289. *parC* mutations were rare and found in only two isolates. In *parE*, Asp585Asn appeared in 30 isolates (31.2%), with Glu596Gln and Phe564Ile identified in a few additional strains. Overall, 68 isolates (70.8%) carried at least one QRDR alteration.

**TABLE 4:** Distribution of tetracycline resistance genes among the 191 *Elizabethkingia* spp. tested in this study

Isolate no.	Species	Tigecycline	Minocycline (With other non-Enterobacterales’s criteria)	Minocycline (with *Stenotrophomonas maltophilia*’s criteria)	Tetracycline resistance gene
9	*E. anophelis*	R	S	S	*tet*(X)
11	*E. anophelis*	I	S	S	*tet*(K)
16	*E. anophelis*	R	S	S	*tet*(X)
23	*E. anophelis*	R	S	I	*tet*(X)
32	*E. anophelis*	R	S	I	*tet*(X)
34	*E. anophelis*	R	S	S	*tet*(X)
39	*E. ursingii*	R	S	S	*tet*(X)
44	*E. miricola*	R	S	S	*tet*(X)
52	*E. anophelis*	R	S	I	*tet*(X)
54	*E. anophelis*	S	S	S	*tet*(K)
67	*E. anophelis*	R	S	S	*tet*(X)
80	*E. miricola*	R	S	S	*tet*(X)
90	*E. anophelis*	R	S	I	*tet*(X)
100	*E. anophelis*	R	S	I	*tet*(X)
101	*E. anophelis*	R	S	I	*tet*(X)
111	*E. anophelis*	R	S	I	*tet*(X)
114	*E. anophelis*	R	S	I	*tet*(X)
116	*E. anophelis*	R	S	I	*tet*(X)
135	*E. anophelis*	R	S	S	*tet*(X)
136	*E. anophelis*	R	S	S	*tet*(X)
152	*E. anophelis*	R	S	S	*tet*(X)
191	*E. anophelis*	R	S	I	*tet*(X)
192	*E. anophelis*	R	S	R	*tet*(X)
194	*E. meningoseptica*	I	S	S	*tet*(X)

Comparison of ciprofloxacin-nonsusceptible isolates that differed in levofloxacin susceptibility (36 susceptible vs 60 nonsusceptible) showed clear contrasts (Table 5). Ser83 substitutions in *gyrA* appeared in 44 of 60 levofloxacin-nonsusceptible isolates (73.3%) but in none of the susceptible isolates (*P* < 0.0001). A similar association was observed in *parE*, where Asp585 substitutions occurred in 29 of 60 nonsusceptible isolates (48.3%) and in only one susceptible isolate (2.8%) (*P* < 0.0001). No consistent pattern was noted in *gyrB* or *parC*. Concurrent *gyrA* and *parE* substitutions were present in 33 isolates, and 32 of them showed resistance to both ciprofloxacin and levofloxacin (data not shown), suggesting that combined mutations may strengthen fluoroquinolone resistance.

**TABLE 5:** Contributions of QRDR profiles to levofloxacin susceptibility among ciprofloxacin-nonsusceptible *Elizabethkingia* spp.

Gene; position of substitution	Ciprofloxacin-nonsuceptible isolates (*n* = 96)^*a*^		
	Levofloxacin-susceptible (*n* = 36)	Levofloxacin-nonsusceptible (*n* = 60)	*P*
*gyrA*			
Wild type	31 (86.11)	14 (23.33)	****
S83	0 (0)	44 (73.33)	****
D87	0 (0)	1 (1.67)	ns^*b*^
N200	1 (2.78)	0 (0)	ns
E207	1 (2.78)	0 (0)	ns
H211	1 (2.78)	0 (0)	ns
D216	2 (5.56)	1 (1.67)	ns
F217	3 (8.33)	3 (5)	ns
*gyrB*			
Wild type	28 (77.78)	48 (80)	ns
G283	2 (5.56)	2 (3.33)	ns
G284	7 (19.44)	6 (10)	ns
G289	2 (5.56)	1 (1.67)	ns
*parC*			
Wild type	35 (97.22)	58 (96.67)	ns
D210	0 (0)	1 (1.67)	ns
S212	1 (2.78)	0 (0)	ns
*parE*			
Wild type	33 (91.67)	24 (40)	****
F564	0 (0)	1 (1.67)	ns
D585	1 (2.78)	29 (48.33)	****
E596	0 (0)	2 (3.33)	ns

^*a*^ The only *E. ursingii* isolate was excluded in this table, due to the limited sample number.

^*b*^ ns, not significant.

### Tetracycline-resistant mechanisms

The detection of tetracycline resistance genes among the 191 isolates revealed the presence of two primary gene types, *tet*(X) and *tet*(K)(Table 4). In this collection, 22 isolates harbored *tet*(X) (22/191, 11.5%) and 2 isolates carried *tet*(K) (2/191, 1.0%). Despite the presence of these resistance genes, their correlation with phenotypic resistance was not uniform across the tetracycline class. Most *tet*(X)-positive isolates exhibited resistance to tigecycline (21/22, 95.5%) and intermediate to tigecycline (1/22, 4.5%), but they all remained susceptible to minocycline. However, with the criteria of *S. maltophilia*, 10 *tet*(X)-positive isolates (10/22, 45.5%) showed non-susceptibility to minocycline, implying that *tet*(X) may contribute to the elevated MIC of minocycline. Both *tet*(K)-carrying isolates showed intermediate to susceptible profiles to tigecycline and complete susceptibility to minocycline, indicating limited clinical relevance of *tet*(K) in *Elizabethkingia*. With the criteria of *S. maltophilia*, a total of 42 isolates (22.0%) were nonsusceptible to minocycline, with 10 isolates harboring the *tet*(X) gene. These findings collectively imply that while *tet*(X) may play a role in the elevated tigecycline non-susceptibility rates and minocycline MICs, while *tet*(K) likely exerts a minor effect on tetracycline-class resistance in this genus (Table 4). When grouped by the presence of *tet* genes (Table S5), *tet(X*)-positive isolates showed high resistance to tigecycline, with both MIC_50_ and MIC_90_ values >8 µg/mL (range: 4 to >8 µg/mL). For *tet*-negative isolates, the MIC_50_ and MIC_90_ were 4 and >8 µg/mL, respectively (range: 1 to >8 µg/mL). Regarding minocycline, *tet(X*)-positive isolates had MIC_50_ and MIC_90_ values of 1 and 2 μg/mL, while *tet*-negative isolates showed slightly lower values of 0.5 and 2 μg/mL. These results highlight a clear difference in the MIC distributions of tigecycline and minocycline between *tet(X*)-positive and *tet*-negative isolates.

## DISCUSSION

Across *Elizabethkingia*, comparative genomics has highlighted a broad accessory virulome that includes adhesins, biofilm loci, RTX-like toxins, and multiple metal acquisition systems, consistent with survival in hospital water systems and immune-compromised hosts (3, 14, 20). In particular, a comparative genomic analysis of *E. meningoseptica* delineated the presence (or strategic absence) of genes involved in sialic acid synthesis, uptake/transport, and catabolism, including *neuC/neuC2* (biosynthesis), *nagB, glmS, glmM* (precursor and amino-sugar interconversion), and sialidase/transport modules, underscoring how *Elizabethkingia* may exploit host sialoglycans at mucosal surfaces (20). The biological importance of these pathways is well established in other pathogens: sialylation and sialic-acid scavenging can promote immune evasion, epithelial adherence, nutrient acquisition, and persistence at host interfaces (23–25), contributing to the organism’s ability to cause severe bacteremia and pneumonia (17, 21, 22).

Previous reports in Taiwan have described the narrow range of therapeutic options for *Elizabethkingia* infections, particularly the consistently poor performance of β-lactams and the uneven activity of several non-β-lactam agents (3, 7, 21, 22, 26). Although the intrinsic β-lactamase genes were not detected by PCR in a few isolates, this might be due to sequence variation at the primer binding sites. Further study using whole-genome sequencing would help clarify the complete genetic background of these specific strains. Fluoroquinolone susceptibility has varied among centers, with ciprofloxacin typically falling between 0% and 13% and levofloxacin around 50%–60%, while minocycline has remained the most reliable agent, maintaining 91%–100% susceptibility in most previous works (3, 22, 26). Tigecycline susceptibility has been notably inconsistent, ranging from only 1.6% in one northern cohort to roughly 24%–60% in earlier surveillance studies (7, 26). Rifampin activity has also differed by region, reaching 90.5% in a northern hospital but dropping substantially in other settings (7). In our analysis, several tendencies echoed these earlier findings, though not uniformly. The ciprofloxacin susceptibility rate (49.2%) was higher than those documented in prior Taiwanese cohorts (7, 22, 26), whereas levofloxacin susceptibility was generally comparable to previous observations (3, 22). Rifampin activity in our isolates was considerably lower (19.9%) than the report in the Northern Taiwan (7), underscoring the extent of regional or temporal variability. Tigecycline activity remained modest at 23% (3, 7, 26). Minocycline retained full susceptibility in our collection, though we did observe isolates with borderline MICs at 4 µg/mL, raising the question of how different breakpoint criteria may alter clinical interpretation. Together, these observations reinforce the need for continued, site-specific antimicrobial surveillance and careful selection of therapeutic agents for *Elizabethkingia* infections (21, 22).

Fluoroquinolone resistance in *Elizabethkingia* species is primarily attributed to chromosomal mutations within QRDRs of *gyrA, gyrB, parC,* and *parE* genes (3); contributions of efflux pumps have been postulated but appear secondary in most clinical sets (27). In the previous study conducted in Taiwan, *gyrA* substitutions at position Ser83 in *E. anophelis* and *E. meningoseptica* were documented, which may reduce fluoroquinolone susceptibility (3). A significant nonsusceptibility to levofloxacin was observed when *gyrA* Ser83 was mutated. In another study, 115 isolates of *E. anophelis* were examined, and 98 were found with mutations at position Ser83 (85%), where the 98 isolates displayed high MIC values to levofloxacin (14). In our study, 96 ciprofloxacin-nonsusceptible isolates underwent QRDR sequencing (Table 6). The canonical hotspot (Ser83 family) was identified 42 isolates (43.8%, 42/96). Notably, a *parE* mutation at Asp585 was also observed amongst our bacterial collection. Further investigation on the contribution of QRDR amino acid substitutions to levofloxacin resistance was conducted, showing a significant correlation of Ser83 in GyrA and Asp585 in ParE to levofloxacin resistance (both *P* < 0.0001). Moreover, the two amino acid substitutions mostly appeared concurrently (data not shown). Additionally, fluoroquinolone resistance in Gram-negative bacteria frequently involves efflux systems (28), particularly the RND family, which lower drug concentrations inside the cell. These pumps can work alone or in combination with target-site mutations to increase MIC values. In many cases, efflux activity explains resistance in strains that lack traditional QRDR mutations, which likely accounts for the 29.2% of non-susceptible isolates observed in our study. Despite their clinical importance in other bacteria, the specific roles and regulation of efflux pumps in *Elizabethkingia* species remain poorly understood and have not been deeply investigated. To sum up, incorporating targeted QRDR genotyping into complex cases (e.g., salvage therapy, refractory bacteremia) may sharpen drug selection.

**TABLE 6:** QRDR profiles of ciprofloxacin-nonsusceptible *Elizabethkingia* spp.

QRDR	*n*/*N* (%)			
	*E. anophelis* (83)	*E. meningseptica* (7)	*E. miricola* (6)	Total (96)
*gyrA*				
Wild type	37 (44.6)	3 (42.9)	5 (83.3)	45 (46.9)
S83I	33 (39.8)	3 (42.9)	0 (0)	36 (37.5)
S83R	6 (7.2)	0 (0)	0 (0)	6 (6.3)
D87Y	1 (1.2)	0 (0)	0 (0)	1 (1)
S83I F217P	1 (1.2)	0 (0)	0 (0)	1 (1)
S83I D216A F217P	1 (1.2)	0 (0)	0 (0)	1 (1)
N200D E207D	0 (0)	0 (0)	1 (16.7)	1 (1)
H211Q D216A	1 (1.2)	0 (0)	0 (0)	1 (1)
D216A F217S	1 (1.2)	0 (0)	0 (0)	1 (1)
F217P	1 (1.2)	1 (14.3)	0 (0)	2 (2.1)
F217S	1 (1.2)	0 (0)	0 (0)	1 (1)
*gyrB*				
Wild type	67 (80.7)	6 (85.7)	3 (50)	76 (79.2)
G284S	9 (10.8)	0 (0)	1 (16.7)	10 (10.4)
G283E	3 (3.6)	0 (0)	0 (0)	3 (3.1)
G283R G284S	1 (1.2)	0 (0)	0 (0)	1 (1)
G284S G289D	2 (2.4)	0 (0)	0 (0)	2 (2.1)
G289D	1 (1.2)	0 (0)	0 (0)	1 (1)
PCR failed	0 (0)	1 (14.3)	2 (33.3)	3 (3.1)
*parC*				
Wild type	81 (97.6)	7 (100)	5 (83.3)	93 (96.9)
D210E	1 (1.2)	0 (0)	0 (0)	1 (1)
S212P	1 (1.2)	0 (0)	0 (0)	1 (1)
PCR failed	0 (0)	0 (0)	1 (16.7)	1 (1)
*parE*				
WT	48 (57.8)	6 (85.7)	3 (50)	57 (59.4)
D585N	29 (34.9)	1 (14.3)	0 (0)	30 (31.2)
E596Q	2 (2.4)	0 (0)	0 (0)	2 (2.1)
F564I	1 (1.2)	0 (0)	0 (0)	1 (1)
PCR failed	3 (3.6)	0 (0)	3 (50)	6 (6.3)

Recent genomic and epidemiological studies indicate that *tet*(X) and its orthologues have a complex evolutionary trajectory with clear potential for cross-species dissemination, which is relevant to our identification of *tet*(X) in clinical *Elizabethkingia* isolates. Members of the *Flavobacteriaceae* are considered an ancestral reservoir of *tet*(X)-type genes (29), while multiple variants, including *tet*(X3), *tet*(X4), and *tet*(X5), have diversified in *Acinetobacter* spp., often located on plasmids or IS-associated transposons that enhance mobility (30). Poirel et al. identified IS*CR2* as a powerful mobile genetic element capable of capturing various resistance genes and promoting multidrug resistance (31). Recent studies suggest that the rapid spread of *tet*(X4) is largely driven by its association with these elements (32), especially IS*CR2*. Through rolling-circle transposition, IS*CR2* helps integrate *tet*(X) into different plasmid backbones (33), forming highly transferable genetic units. This significantly increases the potential for *tet*(X) to spread across different bacterial species, which is a major public health concern as it accelerates the global spread of tigecycline resistance. Plasmid-mediated *tet*(X) variants have also been reported in Enterobacterales and in animal reservoirs, supporting the possibility of cross-sector transmission (34). Environmental surveillance has further documented recombinant *tet*(X) variants in *E. meningoseptica* from aquaculture settings, suggesting that *Elizabethkingia* may act as an intermediate carrier linking environmental and clinical niches (18). Functionally, *tet*(X) encodes a flavin-dependent monooxygenase capable of enzymatically inactivating tigecycline and related glycylcyclines though the phenotypic impact varies with genomic context and expression level (30). This variability is reflected in our findings: *tet*(X)-positive isolates showed consistent resistance to tigecycline but largely retained minocycline susceptibility, consistent with reports that certain *tet*(X) variants preferentially affect glycylcyclines rather than older tetracyclines (18, 29, 30). Interpretation also depends on breakpoint selection; applying the *S. maltophilia* criteria would reclassify nearly half of our *tet*(X)-positive isolates as minocycline-nonsusceptible, underscoring the clinical implications of threshold choice. Overall, these studies support that *Elizabethkingia* may serve as a bridge for mobilizable *tet*(X) genes (18, 29, 30) and that *tet*(X) presence does not uniformly predict resistance across all tetracycline subclasses (30, 34). However, further genetic investigation is needed to fully characterize its mobility and confirm its potential for horizontal transfer. The *tet*(K) determinant, by contrast, is traditionally associated with gram-positive organisms such as staphylococci (35, 36) and is rarely found in gram-negative bacteria (37). Its detection in two of our isolates suggests recent horizontal acquisition and warrants further investigation.

Our study shows some limitations: First, we used breakpoints from other species as guidelines for *Elizabethkingia* are not yet available; second, since the data are from a single medical center, it may not fully represent resistance patterns throughout Taiwan; third, the very small number of rare species, such as *E. ursingii*, makes it difficult to draw broad conclusions about their clinical significance; finally, we did not experimentally investigate the role of efflux pumps in quinolone resistance. Even with these limitations, this work offers a valuable long-term look at the emergence of *tet*(X) and resistance trends in these pathogens.

In conclusion, this longitudinal investigation confirmed *E. anophelis* as the predominant clinical species, consistent with most reports from Taiwan. The antimicrobial susceptibility results highlight severe therapeutic limitations, as none of the 19 agents tested showed consistent activity. Levofloxacin exhibited moderate susceptibility (68.6%), ciprofloxacin demonstrated approximately 50% susceptibility, and minocycline showed 100% susceptibility, underscoring the restricted treatment options for *Elizabethkingia* infections. QRDR sequencing revealed that mutations in *gyrA* and *parE* contributed to fluoroquinolone resistance, whereas our molecular survey identified *tet*(X) and *tet*(K) as emerging tetracycline-resistance determinants. Notably, *tet*(X) carriage correlated with tigecycline non-susceptibility but not with minocycline resistance, suggesting a selective impact on glycylcyclines. To our knowledge, this study provides the first evidence of *tet*-associated genes in clinical *Elizabethkingia* isolates from Taiwan, underscoring the need for continued molecular surveillance and stewardship efforts to limit further dissemination of these multidrug-resistance mechanisms.

## MATERIALS AND METHODS

### Bacterial collection

The study used de-identified bacterial isolates and anonymized clinical data collected retrospectively. Because there was no direct patient contact and no identifiable personal information was accessed, the requirement for formal informed consent was waived. A total of 191 non-duplicate *Elizabethkingia* isolates from blood and sterile sites were collected from patients at the National Taiwan University Hospital (NTUH), a 2,600-bed tertiary referral medical center in Taipei, Taiwan, between January 2016 and December 2022. Each isolate represented a single patient episode to avoid duplication. Species identification was performed using 16S rRNA gene sequencing according to a previous study (38) in distinguishing *Elizabethkingia* species. All isolates were preserved at −80°C in tryptic soy broth supplemented with 20% glycerol until further analysis.

### Antimicrobial susceptibility testing

Antimicrobial susceptibility of the 191 isolates was determined by the agar dilution method, following the Clinical and Laboratory Standards Institute (CLSI) guidelines (Table S1). The testing ranges for each antibiotic are also provided in Table S1. Nineteen antimicrobial agents were tested, including ceftazidime, cefepime, imipenem, meropenem, piperacillin-tazobactam, gentamicin, amikacin, ciprofloxacin, levofloxacin, minocycline, tigecycline, vancomycin, rifampin, sulfamethoxazole-trimethoprim, linezolid, fosfomycin, daptomycin, erythromycin, and colistin. *Escherichia coli* ATCC 25922 and *Pseudomonas aeruginosa* ATCC 27853 were employed for quality control in each experimental batch.

For interpretive criteria, the absence of specific breakpoints for *Elizabethkingia* spp. required the use of surrogate standards. Most agents were interpreted according to CLSI guidelines for Enterobacterales, while selected drugs were assessed using criteria established for non-Enterobacterales or *Enterococcus* spp. (Table S1). For minocycline, we used two CLSI breakpoint sets: Enterobacterales (*S* ≤ 4, *I* = 8, *R* ≥ 16 µg/mL) and *Stenotrophomonas maltophilia* (*S* ≤ 1, *I* = 2, *R* ≥ 4 µg/mL) (Table S1).

### PCR detection

The 16S rRNA gene was amplified with primers listed in Table S2 (38). Sequencing was performed by Genomics BioSci & Tech Co., Ltd. (Taipei, Taiwan). The resulting sequences were analyzed using the BLAST (National Center for Biotechnology Information, NCBI). We compared our sequences with the 16S rRNA gene sequences of type strains from the List of Prokaryotic names with Standing in Nomenclature (LPSN) database (lpsn.dsmz.de). These included *E. anophelis* R26 (EF426425), *E. meningoseptica* ATCC 13,253 (AJ704540), *E. miricola* DSM 14,571 (MH789417), *E. ursingii* G4122 (MH789420), *E. bruuniana* G0146 (MH789419), *E. argenteiflava* YB22 (KY510834), and *E. occulta* G4070 (MH789418). Phylogenetic trees were built using the neighbor-joining method in MEGA 12 (version 12.1.2).

To investigate intrinsic resistance determinants, three chromosomally encoded β-lactamase genes, *bla*_GOB_, *bla*_B_, and *bla*_CME_ were detected (39, 40), which have been implicated in *Elizabethkingia* antimicrobial resistance. For ciprofloxacin-nonsusceptible isolates, the QRDRs of *gyrA*, *gyrB*, *parC*, and *parE* were amplified and sequenced (Genomics BioSci & Tech Co., Ltd.) (38). The obtained sequences were aligned and compared with reference genomes of *E. anophelis* (NZ_CP023010.2), *E. meningoseptica* (NZ_CP016376.1), and *E. miricola* (CP023746.1) to identify amino acid substitutions potentially associated with fluoroquinolone resistance. Tetracycline resistance genes were screened using a multiplex PCR protocol described by Ng et al. (19). PCR-positive amplicons were sequenced and subsequently compared with the BLAST database to confirm gene identity and assess homology. The potential virulence-associated loci, i.e., genes involved in sialic acid synthesis, transport, and utilization (20), were analyzed via PCR and sequencing, including *neuC*, *neuC2*, *nagB*, *glmS*, and *glmM*. All PCRs were conducted with positive controls.

### Pulsed-field gel electrophoresis

Genomic DNA was embedded in agarose plugs and digested with the restriction enzyme *Xho*I (New England Biolabs, USA) (2). Electrophoresis was performed with a CHEF-DR III system (Bio-Rad Laboratories, Hercules, CA, USA). Gel images were captured under UV light and analyzed using BioNumerics software version 6.5 (Applied Maths, Belgium). Dendrograms were constructed based on the Dice similarity coefficient with 1.5% optimization and 1.5% band position tolerance. Clusters were defined using the unweighted pair group method with arithmetic mean (UPGMA). Isolates sharing ≥80% similarity were considered clonally related.

### Statistical analysis

Antimicrobial susceptibility data were summarized in a heatmap generated using R software (version 4.2.2; R Foundation for Statistical Computing, Vienna, Austria). Rows were annotated by *Elizabethkingia* species, number of detected virulence-associated genes, and the presence of intrinsic β-lactamase genes (*bla*_GOB_, *bla*_B_, and *bla*_CME_). Temporal trends in antimicrobial resistance were visualized for five selected antibiotics, ciprofloxacin, levofloxacin, tigecycline, minocycline, and rifampin, using GraphPad Prism version 10 (GraphPad Software, San Diego, CA, USA). For QRDR analysis, Fisher’s exact test was applied to assess the association between amino acid substitutions at specific sites in *gyrA*, *gyrB*, *parC*, and *parE* and levofloxacin resistance among ciprofloxacin nonsusceptible isolates. A two-tailed *P* value of < 0.05 was considered statistically significant.

## SUPPLEMENTAL MATERIAL

Fig. S1 aac.00387-26-s0001.tif 16S rRNA gene phylogenetic trees.

Fig. S2 aac.00387-26-s0002.jpg Dendrogram of the XhoI PFGE results for *E. anophelis* isolates.

Fig. S3 aac.00387-26-s0003.tif Dendrogram of the XhoI PFGE results for *E. meningoseptica* isolates.

Fig. S4 aac.00387-26-s0004.tif Dendrogram of the XhoI PFGE results for *E. miracola* isolates.

Supplemental material aac.00387-26-s0005.docx Supplemental figure legends.

Supplemental tables aac.00387-26-s0006.docx Tables S1 to S5.

Table S6 aac.00387-26-s0007.xlsx The number of base substitutions per site between sequences is shown.

ASM does not own the copyrights to Supplemental Material that may be linked to, or accessed through, an article. The authors have granted ASM a non-exclusive, world-wide license to publish the Supplemental Material files. Please contact the corresponding author directly for reuse.
