# Supplementary figures and images for: Molecular epidemiology and emerging tet(X)-associated resistance of Elizabethkingia spp. in Taiwan, 2016–2022
Source: Antimicrob Agents Chemother. 2026 Jun 12;70(7):e00387-26. doi: 10.1128/aac.00387-26 (PMC13321791; doi:10.1128/aac.00387-26)

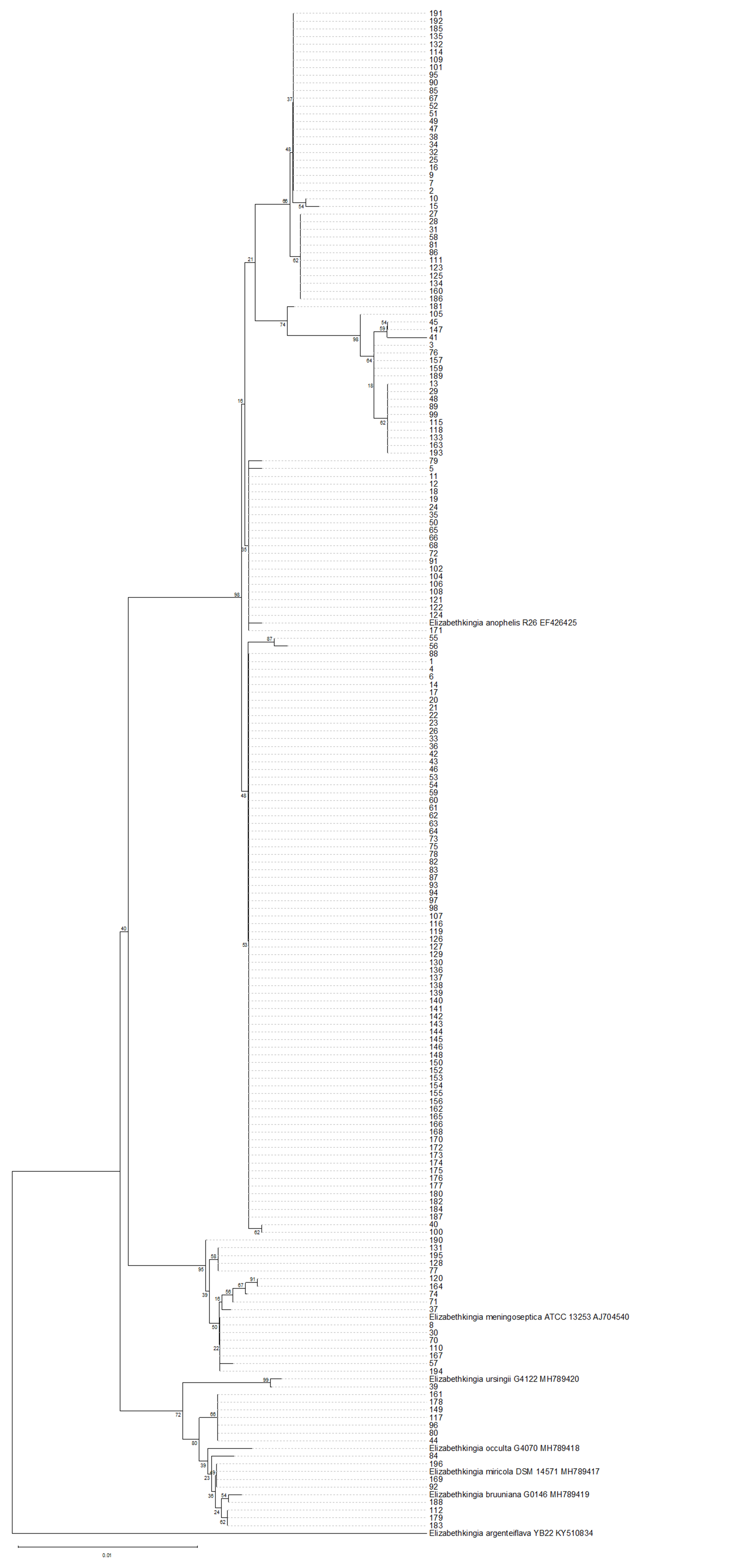

Supplement: Fig. S1 — 16S rRNA gene phylogenetic trees. [file aac.00387-26-s0001.tif]

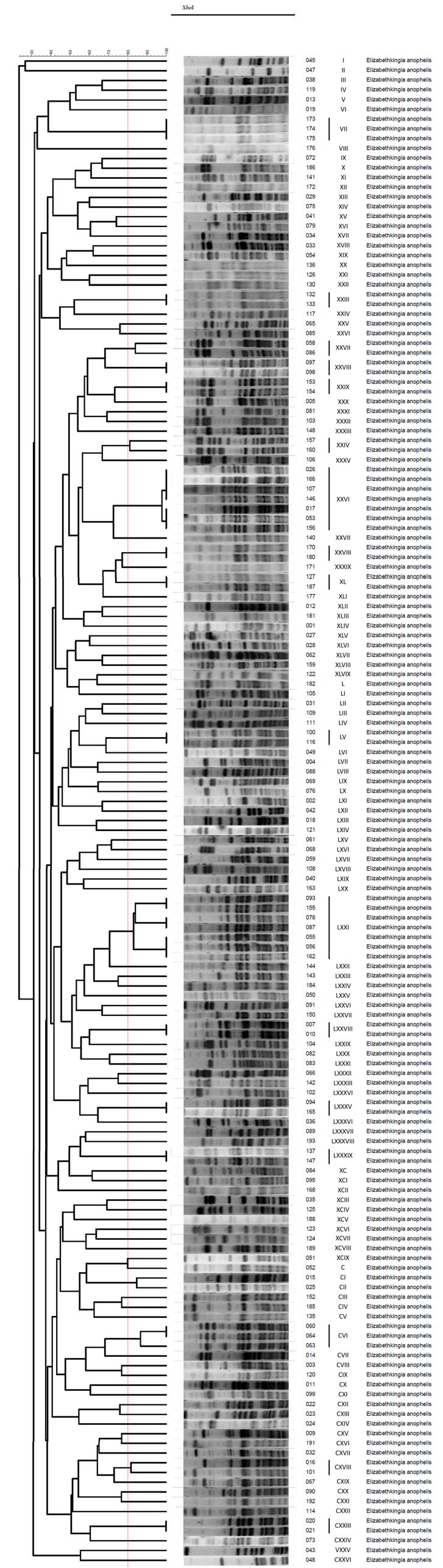

Supplement: Fig. S2 — Dendrogram of the XhoI PFGE results for E. anophelis isolates. [file aac.00387-26-s0002.jpg]

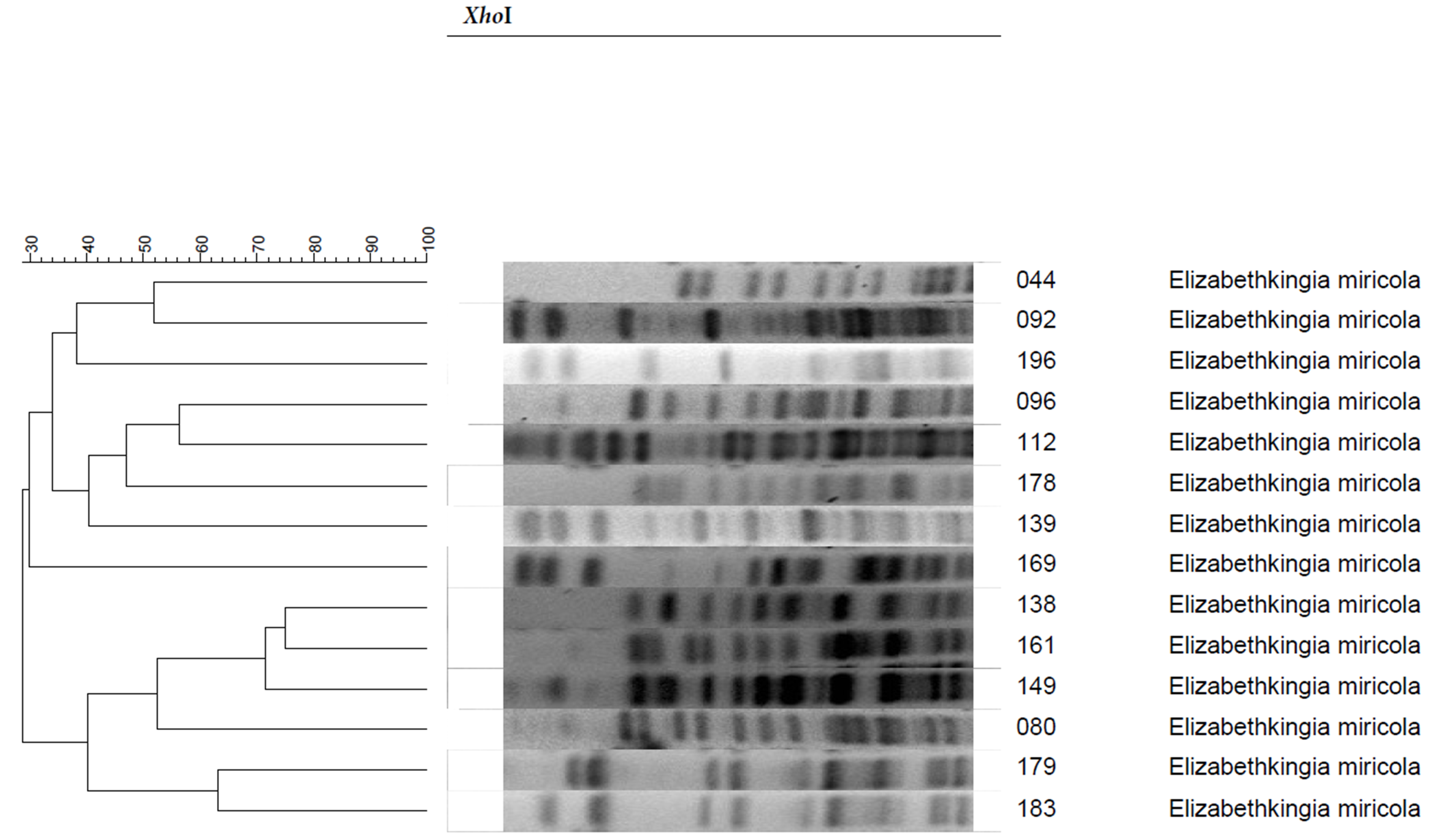

Supplement: Fig. S3 — Dendrogram of the XhoI PFGE results for E. meningoseptica isolates. [file aac.00387-26-s0003.tif]
