# Supplementary material for: Molecular epidemiology and emerging tet(X)-associated resistance of Elizabethkingia spp. in Taiwan, 2016–2022
Source: Antimicrob Agents Chemother. 2026 Jun 12;70(7):e00387-26. doi: 10.1128/aac.00387-26 (PMC13321791; doi:10.1128/aac.00387-26)
Supplement: Supplemental material — Supplemental figure legends. [file aac.00387-26-s0005.docx]

**Supplementary Figure Legends**

**Figure S1. 16S rRNA gene phylogenetic trees.** Trees were built using the neighbor-joining method in MEGA 12. Numbers at the nodes represent bootstrap values (%) based on 2,000 replicates. The scale bar shows evolutionary distance. Because *E. miricola*, *E. occulta*, and *E. bruuniana* are closely related, estimates of their evolutionary divergence are provided in Table S6, where the distance values represent the differences between our isolates and the reference strains.

**Figure S2. Dendrogram of the *XhoI* PFGE results for *E. anophelis* isolates.** Clusters were defined using an 80% similarity threshold.

**Figure S3. Dendrogram of the *XhoI* PFGE results for *E. meningoseptica* isolates.**

**Figure S4. Dendrogram of the *XhoI* PFGE results for *E. miracola* isolates.**
