# Supplementary material for: Molecular epidemiology and emerging tet(X)-associated resistance of Elizabethkingia spp. in Taiwan, 2016–2022
Source: Antimicrob Agents Chemother. 2026 Jun 12;70(7):e00387-26. doi: 10.1128/aac.00387-26 (PMC13321791; doi:10.1128/aac.00387-26)
Supplement: Supplemental tables — Tables S1 to S5. [file aac.00387-26-s0006.docx]

**Supplementary data**

Table S1. Breakpoints of antibiotics tested in this study.

| Antimicrobial agents | Testing range  (μg/ml) | Interpretation (μg/ml) | | | Breakpoints adopted from |
| --- | --- | --- | --- | --- | --- |
|  |  | Susceptible | Intermediate | Resistant |  |
| Amikacin | 1~64 | ≤16 | 32 | ≥64 | 2024 CLSI, Other non-Enterobacterales |
| Cefepime | 1~64 | ≤8 | 16 | ≥32 | 2024, CLSI, Other non-Enterobacterales |
| Ceftazidime | 1~64 | ≤8 | 16 | ≥32 | 2024, CLSI, Other non-Enterobacterales |
| Ciprofloxacin | 0.25~4 | ≤1 | 2 | ≥4 | 2024, CLSI, Other non-Enterobacterales |
| Colistin | 1~16 | N/A | ≤2 | ≥4 | 2024, CLSI, Enterobacterales |
| Daptomycin | 1~32 | ≤2 | 4 | ≥8 | 2024, CLSI, *Enterococcus* spp. |
| Erythromycin | 1~32 | ≤0.5 | 1~4 | ≥8 | 2024, CLSI, *Enterococcus* spp. |
| Fosfomycin | 1~1024 | ≤64 | 128 | ≥256 | 2024, CLSI, Enterobacterales |
| Gentamicin | 2~16 | ≤4 | 8 | ≥16 | 2024, CLSI, Other non-Enterobacterales |
| Imipenem | 1~16 | ≤4 | 8 | ≥16 | 2024, CLSI, Other non-Enterobacterales |
| Levofloxacin | 0.25~8 | ≤2 | 4 | ≥8 | 2024, CLSI, Other non-Enterobacterales |
| Linezolid | 1~64 | ≤2 | 4 | ≥8 | 2024, CLSI, *Enterococcus* spp. |
| Meropenem | 1~16 | ≤4 | 8 | ≥16 | 2024, CLSI, Other non-Enterobacterales |
| Minocycline | 0.0625~16 | ≤4 | 8 | ≥16 | 2024, CLSI, Other non-Enterobacterales |
| *Minocycline | 0.0625~16 | ≤1 | 2 | ≥4 | 2024, CLSI, *Stenotrophomonas maltophilia* |
| Piperacillin/tazobactam | 16~128 | ≤16/4 | 32/4–64/4 | ≥128/4 | 2024, CLSI, Other non-Enterobacterales |
| Rifampin | 0.0625~16 | ≤1 | 2 | ≥4 | 2024, CLSI, *Enterococcus* spp. |
| Tigecycline | 0.25~8 | ≤0.5 | N/A | >0.5 | 2024, EUCAST, Enterobacterales |
| Trimethoprim/sulfamethoxazole | 2/38~32/608 | ≤2/38 | N/A | ≥4/76 | 2024, CLSI, Other non-Enterobacterales |
| Vancomycin | 4~128 | ≤4 | 8~16 | ≥32 | 2024, CLSI, *Enterococcus* spp. |

Table S2. Primers used in this study.

| Primer name | Primer sequence (5'-3') | Target gene | Positive for | Amplicon Size | Reference |
| --- | --- | --- | --- | --- | --- |
| ***bla* genes** |  |  |  |  |  |
| CME-F | AAGAAAGCCACAGTAGCTGTTTC | *bla*_CME_ | *Elizabethkingia* spp. | 695 | [[1](#_ENREF_1)] |
| CME-R | ACTGCAATTGCATAATGTTTACC |  |  |  |  |
| blaB-F | AACTTCGGAAGGAGGGTTGG | *bla*_B_ |  | 136 |  |
| blaB-R | ATGCCAAACCTGGCTGCTAT |  |  |  |  |
| GOB-F | ATGAGAAATTTTGCTACACTG | *bla*_GOB_ |  | 737 | [[2](#_ENREF_2)] |
| GOB-R | TCGAACTGACTTGCATG |  |  |  |  |
| **Quinolone resistance determinant region** | |  |  |  |  |
| gyrA-E.species-F | AGCCCGTTGTTTAAATCCTGAA | *gyrA* | *Elizabethkingia* spp. | 743 | [[3](#_ENREF_3)] |
| gyrA-E.species-R | CCCTGTTGGGAAGTCTGGTG |  |  |  |  |
| Ea_gyrB-F | ATACGCACGAAGGAGGTACG | *gyrB* | *E. anophelis* | 847 | [[4](#_ENREF_4)] |
| Ea_gyrB-R | CGCTCTTTCTCGTTCCATGC |  |  |  |  |
| Emen_gyrB | FAGCGCGATGATATTCCGGTT |  | *E. meningoseptica*/*E. miracola* | 782 | this study |
| Emen_gyrB | RCCACATCGGCATCGGTCATA |  |  |  |  |
| parC-E.species-F | GCTCAGTATGGCAATGCTAAAA | *parC* | *E. anophelis* | 785 | [[3](#_ENREF_3)] |
| parC-E.species-R | TTGCTCTTACCTTACCGCCG |  |  |  |  |
| parC-E.meningoseptica-F | TGACCGGATCAACCGAAGTC |  | *E. meningoseptica*/*E. miracola* | 814 |  |
| parC-E.meningoseptica-R | CAGGTCGCCTGTTGTTTTGG |  |  |  |  |
| parE-E.species-F | GTATTCAGTTTAAAAGGTAAACC | *parE* | - | - | [[3](#_ENREF_3)] |
| parE-E.anophelis-R | GAATATATTGGGCTTCGACA |  | *E. anophelis* | 694 |  |
| parE-E.meningoseptica-R | ACTGAACTTAGTTTGCCATAAG |  | *E. meningoseptica* | 657 |  |
| parE-E.miricola-R | AGAAATCGACATATTCAGAGGT |  | *E. miracola* | 683 |  |
| **Virulence genes** |  |  |  |  |  |
| neuC-F | TGCACCAACAGAACAATCACA | *neuC* | *Elizabethkingia* spp. | 554 | [[5](#_ENREF_5)]  this study |
| neuC-R | TTGTCTGCTTATTCGTGCCT |  |  |  |  |
| neuC2-F | GYGCTATTCCTGCAAAGAAA | *neuC2* |  | 467 |  |
| neuC2-R | YTGAATTTCWGGRCGCATCT |  |  |  |  |
| nagB-F | ATGAYCTKACRCGTAAAGAT | *nagB* |  | 540 |  |
| nagB-R | TTTTCTTCGCNGGATTTGCT |  |  |  |  |
| glmS-F | ARCAGCCAAAATCAATTCAYGA | *glmS* |  | 281 |  |
| glmS-R | ACCNGACTGAGAAATTGCAA |  |  |  |  |
| glmM-F | YATTTCTGGRATTAGAGGGACAA | *glmM* |  | 996 |  |
| glmM-R | ATAATTCCACCGTTWCCTTCTCC |  |  |  |  |
| **Tetracycline resistance related genes** | |  |  |  |  |
| tet(A)-F | GCTACATCCTGCTTGCCTTC | *tet*(A) | - | 210 | [[6](#_ENREF_6)] |
| tet(A)-R | CATAGATCGCCGTGAAGAGG |  |  |  |  |
| tet(B)-F | TTGGTTAGGGGCAAGTTTTG | *tet*(B) |  | 659 |  |
| tet(B)-R | GTAATGGGCCAATAACACCG |  |  |  |  |
| tet(C)-F | CTTGAGAGCCTTCAACCCAG | *tet*(C) |  | 418 |  |
| tet(C)-R | ATGGTCGTCATCTACCTGCC |  |  |  |  |
| tet(D)-F | AAACCATTACGGCATTCTGC | *tet*(D) |  | 787 |  |
| tet(D)-R | GACCGGATACACCATCCATC |  |  |  |  |
| tet(E)-F | AAACCACATCCTCCATACGC | *tet*(E) |  | 278 |  |
| tet(E)-R | AAATAGGCCACAACCGTCAG |  |  |  |  |
| tet(G)-F | GCTCGGTGGTATCTCTGCTC | *tet*(G) |  | 468 |  |
| tet(G)-R | AGCAACAGAATCGGGAACAC |  |  |  |  |
| tet(K)-F | TCGATAGGAACAGCAGTA | *tet*(K) |  | 169 |  |
| tet(K)-R | CAGCAGATCCTACTCCTT |  |  |  |  |
| tet(L)-F | TCGTTAGCGTGCTGTCATTC | *tet*(L) |  | 267 |  |
| tet(L)-R | GTATCCCACCAATGTAGCCG |  |  |  |  |
| tet(M)-F | GTGGACAAAGGTACAACGAG | *tet*(M) |  | 406 |  |
| tet(M)-R | CGGTAAAGTTCGTCACACAC |  |  |  |  |
| tet(O)-F | AACTTAGGCATTCTGGCTCAC | *tet*(O) |  | 515 |  |
| tet(O)-R | TCCCACTGTTCCATATCGTCA |  |  |  |  |
| tet(S)-F | CATAGACAAGCCGTTGACC | *tet*(S) |  | 667 |  |
| tet(S)-R | ATGTTTTTGGAACGCCAGAG |  |  |  |  |
| tetA(P)-F | CTTGGATTGCGGAAGAAGAG | *tet*A(P) |  | 676 |  |
| tetA(P)-R | ATATGCCCATTTAACCACGC |  |  |  |  |
| tet(Q)-F | TTATACTTCCTCCGGCATCG | *tet*(Q) |  | 904 |  |
| tet(Q)-R | ATCGGTTCGAGAATGTCCAC |  |  |  |  |
| tet(X)-F | CAATAATTGGTGGTGGACCC | *tet*(X) |  | 468 |  |
| tet(X)-R | TTCTTACCTTGGACATCCCG |  |  |  |  |
| ISCR2-F | AAGAATTTCTCCAATGCGGG | IS*CR2* |  | 215 | [[7](#_ENREF_7)]  this study |
| ISCR2-R | CTGAAACGGGAAGCTCAACA |  |  |  |  |

**Table S3**. β-lactamase gene detection of *Elizabethkingia* spp.

| **Strain** | **PCR detection, *n/N (%*)** | | | |
| --- | --- | --- | --- | --- |
|  | ***E. anopheles* (156)** | ***E. meningseptica* (19)** | ***E. miricola* (15)** | **Total (191) ^a^** |
| ***bla*_GOB_** | **148 (94.9)** | **7 (36.8)** | **14 (93.3)** | **170 (89.1)** |
| ***bla*_B_** | **152 (97.5)** | **10 (52.6)** | **11 (73.3)** | **174 (91.1)** |
| ***bla*_CME_** | **155 (99.4)** | **16 (84.2)** | **15 (100)** | **187 (97.9)** |

**^a^, one of the isolates we tested is *E. ursingii.***

**Table S4**. Virulence gene detection of *Elizabethkingia* spp.

| **Strain^a^** | **PCR detection, *n/N (%)*** | | | |
| --- | --- | --- | --- | --- |
|  | ***E. anopheles* (156)** | ***E. meningseptica* (19)** | ***E. miricola* (15)** | **Total (191) ^a^** |
| ***glmS*** | **150 (96.2)** | **19 (100)** | **8 (53.3)** | **178 (93.2)** |
| ***glmM*** | **152 (97.5)** | **19 (100)** | **14 (93.3)** | **186 (97.4)** |
| ***nagB*** | **153 (98.1)** | **19 (100)** | **15 (100)** | **188 (98.4)** |
| ***neuC2*** | **31 (20.9)** | **9 (47.4)** | **5 (33.3)** | **46 (24.5)** |
| ***neuC*** | **0 (0)** | **4 (21.1)** | **0 (0)** | **4 (2.1)** |

**^a^, one of the isolates we tested is *E. ursingii.***

**Table S5**. MIC distributions of tigecycline and minocycline against 191 *Elizabethkingia* isolates stratified by *tet* genes.

| Group | Tigecycline | | |  | Minocycline | | |
| --- | --- | --- | --- | --- | --- | --- | --- |
|  | MIC_50_ (μg/ml) | MIC_90_ (μg/ml) | MIC range (μg/ml) |  | MIC_50_ (μg/ml) | MIC_90_ (μg/ml) | MIC range (μg/ml) |
| All (n=191) | 4 | >8 | 1~>8 |  | 1 | 2 | 0.125~4 |
| *tet*(X)-positive (n=22) | >8 | >8 | 4~>8 |  | 1 | 2 | 1~4 |
| *tet*(K)-positive (n=2) | 2 | 4 | 2~4 |  | 0.5 | 1 | 0.5~1 |
| *tet*-negative isolates (n=167) | 4 | >8 | 1~>8 |  | 0.5 | 2 | 0.125~2 |

**Reference**

1. Gonzalez, L.J. and A.J. Vila, *Carbapenem resistance in Elizabethkingia meningoseptica is mediated by metallo-beta-lactamase BlaB.* Antimicrob Agents Chemother, 2012. **56**(4): p. 1686-92.

2. Colapietro, M., et al., *BlaB-15, a new BlaB metallo-beta-lactamase variant found in an Elizabethkingia miricola clinical isolate.* Diagn Microbiol Infect Dis, 2016. **85**(2): p. 195-7.

3. Jian, M.J., et al., *Molecular typing and profiling of topoisomerase mutations causing resistance to ciprofloxacin and levofloxacin in Elizabethkingia species.* PeerJ, 2018. **6**: p. e5608.

4. Lin, J.N., et al., *Elizabethkingia bruuniana Infections in Humans, Taiwan, 2005-2017.* Emerg Infect Dis, 2019. **25**(7): p. 1412-1414.

5. Chen, S., et al., *Comparative genomic analyses reveal diverse virulence factors and antimicrobial resistance mechanisms in clinical Elizabethkingia meningoseptica strains.* PLoS One, 2019. **14**(10): p. e0222648.

6. Ng, L.K., et al., *Multiplex PCR for the detection of tetracycline resistant genes.* Mol Cell Probes, 2001. **15**(4): p. 209-15.

7. Poirel, L., et al., *ISCR2, another vehicle for bla(VEB) gene acquisition.* Antimicrob Agents Chemother, 2009. **53**(11): p. 4940-3.
